# Supplementary material for: Risk factors of phlebitis in patients admitted to the intensive care unit vary according to the duration of catheter dwelling: A post-hoc analysis of the AMOR-VENUS study
Source: PLoS One. 2025 Apr 30;20(4):e0320583. doi: 10.1371/journal.pone.0320583 (PMC12043149; doi:10.1371/journal.pone.0320583)
Supplement: S2 File — (DOCX) [file pone.0320583.s002.docx]

**Definition and measurement of phlebitis**

A blinded assessor at the main institution diagnosed phlebitis and classified it into four grades based on criteria of six clinical signs (Supplementary table 2-3). For patients with loss of consciousness, well-trained nurses assessed the grade of pain and other symptoms using the Facial and Behavioral Pain Scale ^1^. We conducted pilot training to ensure accurate phlebitis diagnosis and reduce information bias. In addition, well-trained expert clinician-researchers in the central institution monitored the accuracy of phlebitis diagnosis during the study period. During the first month of data collection, the data management center confirmed the accuracy of catheter insertion site information with phlebitis images sent from each facility.

**References**

1. Infusion Nurses Society. Infusion nursing standards of practice. J Infus Nurs (2006) 29:S1-92. doi: 10.1097/00129804-200601001-00001.

**Supplementary table 2. The definition of phlebitis according to the Infusion Nurses Society (INS)**

| Grade | Definition |
| --- | --- |
| 0 | No clinical signs. |
| 1 | Erythema at punctured site regardless of the presence or absence of pain. |
| 2 | Pain at punctured site with erythema and/or edema. |
| 3 | Pain at punctured site with erythema and/or edema, and streak formation or palpable venous cord. |
| 4 | Pain at punctured site with erythema and/or edema, and streak formation or palpable venous cord > 1 inch. Presence of purulent drainage. |

**Supplementary table 3. The definition of each variable for phlebitis according to the Infusion Nurses Society (INS)**

| Variable | Definition |
| --- | --- |
| Pain | Pain around PIVC inserted site |
| Erythema | Erythema around PIVC inserted site |
| Edema | Swelling around PIVC inserted site |
| Streak formation | Erythema along blood vessels from PIVC inserted site |
| Palpable venous cord | Induration along blood vessels from PIVC inserted site |

Abbreviation: PIVC, peripheral intravenous catheter.
