# Supplementary material for: Risk factors of phlebitis in patients admitted to the intensive care unit vary according to the duration of catheter dwelling: A post-hoc analysis of the AMOR-VENUS study
Source: PLoS One. 2025 Apr 30;20(4):e0320583. doi: 10.1371/journal.pone.0320583 (PMC12043149; doi:10.1371/journal.pone.0320583)
Supplement: S3 File — (DOCX) [file pone.0320583.s003.docx]

**Presumed risk factors for phlebitis**

Based on a previous study ^1^, the following presumed risk factors for phlebitis were extracted: patient characteristics (age, sex, BMI, and APACHE II score), type of admission to the ICU, PIVC characteristics (provision of standardized drug administration measures in the ICU, medical staff inserting the catheter, insertion site, catheter materials, catheter size), and drugs administered via PIVCs during ICU stay (ampicillin/sulbactam, dexmedetomidine, fat, fentanyl, heparin, midazolam, nicardipine, and noradrenaline). BMI was categorized into three groups based on the World Health Organization classification for the Asian population, as follows: ≤ 18.5, 18.6–25, and > 25 kg/m^2^ ^2^. The drugs included in this model as binary data were based on a previous study ^1^ and were selected for the following reasons: (1) the top six drugs administered more frequently than 5% of all PIVCs, (2) the calculated p-value of phlebitis in multivariate marginal Cox regression analysis of previous studies was less than 0.05, and (3) clinically important. Drugs that could not be stratified due to the small sample of patients receiving these drugs were excluded, and a maximum of eight drugs were selected based on categories (1)–(3).

**References**

1. Yasuda H, Rickard CM, Marsh N, Yamamoto R, Kotani Y, Kishihara Y, et al. Risk factors for peripheral intravascular catheter-related phlebitis in critically ill patients: analysis of 3429 catheters from 23 Japanese intensive care units. Annals of Intensive Care. 2022; 12: 33.
2. Appropriate body-mass index for Asian populations and its implications for policy and intervention strategies. The Lancet. 2004; 363: 157-63.
