# Supplementary material for: Risk factors of phlebitis in patients admitted to the intensive care unit vary according to the duration of catheter dwelling: A post-hoc analysis of the AMOR-VENUS study
Source: PLoS One. 2025 Apr 30;20(4):e0320583. doi: 10.1371/journal.pone.0320583 (PMC12043149; doi:10.1371/journal.pone.0320583)
Supplement: S4 File — (DOCX) [file pone.0320583.s004.docx]

**Supplementary table 4. Univariate analysis with marginal Cox regression analysis for the risk factors of phlebitis stratified by the duration of catheter dwelling**

| **Variables** | **Duration of catheter dwelling groups** | | | | | |
| --- | --- | --- | --- | --- | --- | --- |
|  | **≤ 24 h**  **(n=1,040)**  **Phlebitis: n=103 (9.9%)** | | **>24 h, ≤ 72 h**  **(n=1,324)**  **Phlebitis: n=151 (11.4%)** | | **> 72 h**  **(n=984)**  **Phlebitis: n=49 (5.0%)** | |
|  | **HR (95% CI)** | **p value** | **HR (95% CI)** | **p value** | **HR (95% CI)** | **p value** |
| Age (years) | 1.01 (0.99-1.02) | 0.38 | 1.0 (0.99-1.02) | 0.97 | 1.0 (0.98-1.02) | 0.97 |
| Male sex | 0.93 (0.63-1.39) | 0.73 | 0.53 (0.39-0.73) | < .01 | 0.65 (0.37-1.14) | 0.13 |
| BMI (kg/m^2^)  18.6–25  ≤ 18.5  >25 < | ref  0.9 (0.52-1.59)  0.91 (0.57-1.44) | -  0.73  0.68 | ref  1.14 (0.71-1.83)  0.97 (0.67-1.41) | -  0.59  0.87 | ref  1.34 (0.6-2.94)  1.45 (0.77-2.71) | -  0.47  0.25 |
| APACHE II, score  16–25  ≤15  ≥26 | 0.68 (0.44-1.06)  ref  1.14 (0.69-1.89) | 0.09  -  0.61 | 0.91 (0.64-1.29)  ref  0.58 (0.36-0.93) | 0.59  -  0.02 | 1.28 (0.69-2.36)  ref  0.61 (0.27-1.38) | 0.43  -  0.24 |
| Type of admission to ICU  Elective surgical  Emergency surgical  Non-surgical | ref  3.02 (1.57-5.83)  3.35 (1.91-5.88) | -  < .01  < .01 | ref  2.36 (1.24-4.51)  1.8 (1.01-3.2) | -  < .01  0.05 | ref  1.48 (0.57-3.86)  1.11 (0.46-2.68) | -  0.42  0.81 |
| Provision of standardized drug administration measures in the ICU | 0.26 (0.11-0.65) | < .01 | 0.7 (0.29-1.71) | 0.44 | 0.43 (0.06-3.12) | 0.40 |
| Medical staff inserting the catheter  Nurse  Doctor | ref  1.1 (0.55-2.2) | -  0.78 | ref  0.56 (0.26-1.21) | -  0.14 | ref  0.56 (0.22-1.44) | -  0.23 |
| Insertion site  Forearm  Upper arm  Elbow  Wrist  Hand  Lower leg  Dorsal foot | ref  0.66 (0.3-1.45)  1.73 (0.83-3.63)  0.9 (0.39-2.08)  0.55 (0.29-1.02)  1.45 (0.72-2.93)  1.55 (0.49-4.97) | -  0.3  0.15  0.8  0.06  0.3  0.46 | ref  0.51 (0.28-0.96)  0.22 (0.05-0.88)  0.26 (0.06-1.03)  0.43 (0.23-0.8)  0.76 (0.42-1.38)  1.26 (0.64-2.35) | -  0.04  0.04  0.06  < .01  0.37  0.54 | ref  1.04 (0.36-3.01)  1.28 (0.39-4.26)  1.61 (0.48-5.34)  1.09 (0.52-2.29)  0.30 (0.04-2.22)  1.24 (0.43-3.59) | -  0.94  0.69  0.44  0.82  0.24  0.69 |
| Catheter material  Polyurethane  PEU-Vialon^®^†  Tetrafluoroethylene | ref  0.98 (0.58-1.65)  1.21 (0.76-1.92) | -  0.92  0.43 | ref  0.5 (0.33-0.76)  0.52 (0.36-0.75) | -  < .01  < .01 | ref  0.77 (0.39-1.52)  0.71 (0.34-1.52) | -  0.45  0.38 |
| Catheter size  22–24G  ≥18G  20G | ref  0.44 (0.18-1.1)  0.68 (0.42-1.09) | -  0.08  0.11 | ref  0.6 (0.22-1.62)  0.39 (0.24-0.63) | -  0.31  < .01 | ref  3.76 (1.71-8.29)  1.1 (0.57-2.13) | -  < .01  0.77 |
| Administered drug  Ampicillin/sulbactam  Dexmedetomidine  Fat  Fentanyl  Heparin  Midazolam  Nicardipine  Noradrenaline | 0.74 (0.30-1.82)  2.98 (1.63-5.46)  2.32 (1.38-3.91)  2.59 (1.31-5.14)  1.01 (0.49-2.08)  2.48 (0.91-6.75)  0.69 (0.32-1.48)  2.64 (1.07-6.5) | 0.51  < .01  < .01  < .01  0.98  0.08  0.33  0.03 | 1.41(0.80-2.49)  1.57 (0.98-2.52)  0.91 (0.57-1.44)  0.94 (0.54-1.63)  0.77 (0.39-1.5)  1.53 (0.57-4.12)  3.21 (2.21-4.67)  2.8 (1.47-5.31) | 0.24  0.06  0.68  0.82  0.44  0.4  < .01  < .01 | 0.73(0.18-3.02)  0.79 (0.33-1.85)  0.91 (0.44-1.88)  0.92 (0.44-1.89)  1.03 (0.51-2.07)  1.36 (0.33-5.61)  2.16 (1.01-4.62)  3.0 (0.93-9.67) | 0.67  0.58  0.81  0.81  0.94  0.67  0.05  0.07 |

Abbreviation: APACHE, acute physiology and chronic health evaluation; BMI, body mass index; CI, confidence interval; ER, emergency room; ICU, intensive care unit; IQR, interquartile range; HR, hazard ratio; PIVC, peripheral intravenous catheter

† PEU-Vialon^®^ is specified polyurethane.
