# Supplementary material for: Risk factors of phlebitis in patients admitted to the intensive care unit vary according to the duration of catheter dwelling: A post-hoc analysis of the AMOR-VENUS study
Source: PLoS One. 2025 Apr 30;20(4):e0320583. doi: 10.1371/journal.pone.0320583 (PMC12043149; doi:10.1371/journal.pone.0320583)
Supplement: Attachment — Submitted filename: Response_to_Reviewers.docx [file pone.0320583.s006.docx]

November, 2024
Ioannis Savvas, DVM, Ph.D.
Academic Editor
*PLOS ONE*

Dear Dr. Savvas,

We are pleased to be given an opportunity to resubmit our manuscript titled "Risk factors of phlebitis in patients admitted to the intensive care unit vary according to the duration of catheter dwelling: A post-hoc analysis of the AMOR-VENUS study" for further consideration in *PLOS ONE*. The Manuscript ID is: PONE-D-23-30822.

We would like to thank you and the reviewers for the valuable feedback and insightful suggestions, which have helped us improve the quality of our manuscript. I look forward to working with you and the reviewers to move this manuscript closer to publication in the *PLOS ONE*.

In response to the reviewers' comments, we have made significant revisions, which we believe have strengthened our work. A detailed point-by-point response to each reviewer’s comment is included below. Additionally, the revisions made in the manuscript have been highlighted in the track-changes version.

We hope that the revised manuscript meets your expectations, and we look forward to your editorial decision. Thank you again for considering our work for publication in *PLOS ONE*.

Sincerely,

Hideto Yasuda
Department of Emergency and Critical Care Medicine,
1-847, Amanuma-cho, Omiya-ku, Saitama City,
Saitama, 330-8503, Japan
Phone: +81-(0)48-647-2111
Fax: +81-(0)48-648-5180
Email: [yasudahideto@me.com](mailto:yasudahideto@me.com)

**Our responses to the Reviewers' comments and suggestions are listed below:**

**Reviewer #1**

1. **Comment:**
   *Line 99: What was the purpose of the analysis of variance and Kruskal-Wallis test?*

**Response:**
Thank you for your question. The Kruskal-Wallis test was performed to compare between the three groups in terms of patient background characteristics to assess whether there were significant differences among the groups categorized by catheter insertion time. Our goal was to assess differences in background characteristics that might impact phlebitis risk, rather than to detect pairwise differences between any specific groups.

2. **Comment:**
   *Line 101: What was the purpose of the Fisher’s exact test and Pearson’s chi-square test, as the association of interest is with phlebitis, which is assessed with Cox regression analyses? Clarify if it is to test for differences according to duration of catheter dwelling group. Should the patient and/or hospital effects be considered in these tests? Would a mixed hierarchical model be more appropriate for assessing these differences?*

**Response:**
Thank you for pointing this out. The Fisher’s exact test and Pearson’s chi-square test were used for the univariate analysis to detect differences between the groups rather than to explore associations with phlebitis. These tests aimed to provide an initial understanding of background differences among the groups categorized by catheter dwelling duration. Regarding association testing, we used multilevel Cox regression, which incorporated patient and hospital effects as random effects to account for hierarchical variability. Given the recent advances in analysis techniques, it might indeed have been suitable to omit these background tests entirely, as differences in background characteristics have been less frequently tested in recent literature.

3. **Comment:**
   *Is the endpoint really time to phlebitis from insertion of PIVC (rather than incidence of phlebitis)?*

**Response:**
Thank you for this query. As you kindly pointed out, the endpoint in our study was "time to phlebitis from insertion of PIVC." Although the exact time of phlebitis onset may not have been precisely recorded, we monitored the catheter insertion site every four hours, allowing us to estimate the time to phlebitis onset with reasonable accuracy.

4. **Comment:**
   *How were patient and ICU accounted for in the Cox regression analyses? Should this also be modeled hierarchically?*

**Response:**
Thank you for pointing this out. In our analysis, we used multilevel Cox regression, incorporating both patient and facility (ICU) as random effects to address potential clustering effects. This hierarchical modeling approach allowed us to account for variability in catheter management practices across facilities and among patients who had multiple catheters.

**Reviewer #2**

1. **Comment:**
   *There is little justification for the dichotomization of 24 vs >72 hours.*

**Response:**
Thank you for your comment. We considered categorizing the groups into three distinct categories based on catheter dwelling duration because early infections post-insertion is common in critically ill patients. Although there was no universally accepted cutoff, we selected a 72-hour threshold to achieve a balanced sample size in each group, which could help reduce the risk of overfitting and ensure robust statistical analysis.

2. **Comment:**
   *The biological plausibility of why certain drugs but not others may be associated with later phlebitis is not well explored.*

**Response:**
Thank you for your comment. The factors influencing phlebitis onset, particularly medication effects, remain complex and are not fully understood. The existing studies often treat medication as a binary variable (administered or not), which does not capture the timing or dose effects. Ideally, the time-dependent effects of medication on phlebitis onset would require a more sophisticated analysis. In our study, we did not investigate specific timing due to model simplicity; this limitation is noted for future studies.

3. **Comment:**

*the discrepant findings reported here from their previous paper which found a shorter dwell time with phlebitis positive patients vs phlebitis negative patients (Duration of catheter dwell, median (IQR), hour 37.0 (19.2–57.6) with phlebitis vs 44.8 (21.0–81.5) without phlebitis p < 0.01) is also not discussed.*

**Response:**

Thank you for your suggestion. We agree that an explanation was necessary. Accordingly, we have added an explanation to the manuscript (Lines 216-223).

4. **Comment:**
   *Discrepant findings regarding dwell time and phlebitis. How does the confounder of catheter removal due to suspected phlebitis (with a corresponding shorter dwell time) affect your findings?*

**Response:**
We appreciate your insightful observation. In our study, early catheter removal due to suspected phlebitis might have led to a shorter dwelling duration, potentially impacting the results. If catheter dwelling duration was reduced due to phlebitis occurrence, this might have influenced the observed relationship between phlebitis incidence and dwelling time. Cases of extended dwelling durations could have shown different phlebitis outcomes. This limitation has been noted in our manuscript, as we recognize the need for further research to clarify this aspect. We have added this meaning to the manuscript (Lines 249-255).

**Reviewer #3**

1. **Comment:**
   *Several catheters (2.5 on average) were inserted per patient. How did the authors consider this in their statistical analyses?*

**Response:**
Thank you for your question. Given the possibility that outcomes might be correlated across multiple catheters in the same patient, we performed a multilevel analysis with both patient and facility factors as random effects. This approach helped us account for the nested structure of our data and reduced the risk of inflated associations due to within-patient correlations.

2. **Comment:**
   *Patients with missing data were excluded from the analysis. How many patients/catheters does this represent?*

**Response:**
Thank you for your question. We have added a detailed account of the number of patients and catheters excluded due to missing data. This information is reported in the results section (Line 164-167).

3. **Comment:**
   *Implications for clinical practice: The duration of catheterization is rarely known when the catheter is inserted. How will identifying phlebitis risk factors based on dwell time change practices?*

**Response:**
We appreciate your insightful query. Our study identified that risk factors for phlebitis onset varied with catheterization duration. For instance, in cases of high risk for phlebitis within the first 24 hours, scheduled replacement might be considered within that period. Conversely, if no risk factors appeared until after 72 hours, the catheter could potentially be monitored until then, delaying replacement. This approach could help estimate optimal replacement timing, which we discussed in the manuscript (Line 232-242).

We look forward to your feedback and hope that our revised manuscript is now suitable for publication.
