# Supplementary material for: Risk factors of phlebitis in patients admitted to the intensive care unit vary according to the duration of catheter dwelling: A post-hoc analysis of the AMOR-VENUS study
Source: PLoS One. 2025 Apr 30;20(4):e0320583. doi: 10.1371/journal.pone.0320583 (PMC12043149; doi:10.1371/journal.pone.0320583)
Supplement: Attachment — Submitted filename: Response_to_Reviewers_auresp_2.docx [file pone.0320583.s007.docx]

February, 2025
Ioannis Savvas, DVM, Ph.D.
Academic Editor
*PLOS ONE*

Dear Dr. Savvas,

We are pleased to be given an opportunity to resubmit our manuscript titled "Risk factors of phlebitis in patients admitted to the intensive care unit vary according to the duration of catheter dwelling: A post-hoc analysis of the AMOR-VENUS study" for further consideration in *PLOS ONE*. The Manuscript ID is: PONE-D-23-30822.

We would like to thank you and the reviewers for the valuable feedback and insightful suggestions, which have helped us improve the quality of our manuscript. I look forward to working with you and the reviewers to move this manuscript closer to publication in the *PLOS ONE*.

In response to the reviewers' comments, we have made substantial and detailed revisions to comprehensively respond to all comments. Specifically, we have:

- Elaborated on our statistical methodology, explicitly explaining the rationale for each test used.
- Refined the definition of the study endpoint, ensuring clarity regarding how "time to phlebitis" was determined.
- Justified the use of hierarchical modeling, with a clear explanation about why this approach was necessary.
- Expanded the discussion about clinical implications, outlining how our findings could help establish catheter management strategies in intensive care settings.

We sincerely appreciate the reviewers' valuable insights, which have helped us significantly improve our manuscript. For clarity, all revisions have been highlighted in the track-changes version of the manuscript.

We hope that the revised manuscript meets your expectations; we look forward to your editorial decision. Thank you once again for considering our work for publication in *PLOS ONE*.

Sincerely,

Hideto Yasuda
Department of Emergency and Critical Care Medicine,
1-847, Amanuma-cho, Omiya-ku, Saitama City,
Saitama, 330-8503, Japan
Phone: +81-(0)48-647-2111
Fax: +81-(0)48-648-5180
Email: [yasudahideto@me.com](mailto:yasudahideto@me.com)

**Our responses to the Reviewers' comments and suggestions are listed below:**

**Reviewer #1**

1. **Comment:**

*There was no text change to address the four points that I made in the comments - thus another reader may experience the same confusion as I. Please add some important words to the manuscript to clarify these points.*

**Response:** We thank you for your valuable and insightful feedback. **We sincerely apologize for any lack of clarity in our previous revision** and have now made the necessary modifications to explicitly respond to all four comments. The following revisions have been made to ensure the manuscript is clearer and more comprehensive.

*1. Line 99: What was the purpose of the analysis of variance and Kruskal-Wallis test?*

**Response:**
Thank you for your question. The **analyses of variance (ANOVA) and Kruskal-Wallis test were used to compare background characteristics** among the three catheter insertion time groups to determine whether significant differences existed. **This was necessary for confirming that the groups were comparable before performing further statistical analyses.** We have clarified this in the Methods section (Lines 100-102).

**2.***Line 101: What was the purpose of the Fisher’s exact test and Pearson’s chi-square test, as the association of interest is with phlebitis, which is assessed with Cox regression analyses? Clarify if it is to test for differences according to duration of catheter dwelling group. Should the patient and/or hospital effects be considered in these tests? Would a mixed hierarchical model be more appropriate for assessing these differences?*

**Response:**
Thank you for pointing this out. **In the revised manuscript, we have explicitly stated that Fisher’s Exact Test and Pearson’s Chi-Square Test were used solely for preliminary analyzing background characteristics** and were **not intended to infer causal relationships with phlebitis risk.**
Furthermore, **we have emphasized that hierarchical modeling (multilevel Cox regression) was applied in the main analysis to appropriately account for both patient- and ICU-level effects.** This would ensure that confounding due to clustering at the institutional and patient levels was minimized (Lines 104-108).

**3.***Is the endpoint really time to phlebitis from insertion of PIVC (rather than incidence of phlebitis)?*

**Response:**
Thank you for your insightful query. **We have now explicitly clarified in the Methods section that the study endpoint was "time to phlebitis from insertion of PIVC," as determined based on regular 4-hour monitoring.**
This approach ensures that **the earliest possible detection of phlebitis onset** is made while maintaining consistency in timepoint evaluation across patients (Lines 113-115).

**4.***How were patient and ICU accounted for in the Cox regression analyses? Should this also be modeled hierarchically?*

**Response:**
Thank you for pointing this out. We used multilevel Cox regression, incorporating both patient and facility (ICU) as random effects to address potential clustering effects. In the revised manuscript, we have now explicitly justified our choice of this approach, ensuring clarity on why hierarchical modeling was necessary (Lines 106-108).

We sincerely appreciate the time and effort invested by the reviewers and editors for evaluating our manuscript. We believe that these revisions have significantly strengthened our study and ensured its clarity, rigor, and clinical relevance.
We **look forward to your feedback and hope that our revised manuscript now fully meets the criteria for publication in PLOS ONE.**
